# Habitat Characteristics and Mineral Nutrition Status of *Rubus chamaemorus* L. in Latvia

**DOI:** 10.3390/plants12030528

**Published:** 2023-01-24

**Authors:** Laura Āboliņa, Anita Osvalde, Andis Karlsons

**Affiliations:** 1 Institute of Biology, University of Latvia, LV-1004 Rīga, Latvia; 2 Faculty of Agriculture, Latvia University of Life Sciences and Technologies, LV-3001 Jelgava, Latvia

**Keywords:** cloudberry, surrounding species composition, soil analysis, leaf testing, sphagnum moss, peat bogs

## Abstract

In Latvia, cloudberries are considered a valuable delicacy and have aroused interest in the possibility of commercial cultivation, as currently, they are collected only in the wild. A complex study was carried out to provide insight into the growth conditions of wild cloudberry in Latvia. The knowledge gained would provide a basis for the development of cloudberry cultivation technologies in the hemiboreal zone. Habitat characteristics, composition of surrounding vegetation, and plant mineral nutrition status were investigated in 18 study sites. Overall, the species composition of cloudberry study sites corresponded to two plant community classes: Cl. *Vaccinio-Piceetea* and Cl. *Oxycocco-Sphagnetea*. The most common species were *Sphagnum magellanicum, Vaccinium myrtillus*, and *Oxycoccus palustris*. The results clearly indicated acidic peat soils with high organic matter content and low degree of decomposition as being most suitable for cloudberry cultivation. High nutrient uptake capacity was found for wild cloudberry growing in nutrient-poor environments, as most of the leaf nutrients corresponded to the optimal levels determined for different cultivated berries. However, balanced fertilization to ensure successful plant vegetative and root growth would be recommended. The first results on wild cloudberry in Latvia indicated that optimization of P, S, B, and Mo should be the main focus.

## 1. Introduction

In Europe, interest in wild products is growing, showing a renewed interest in the ecosystem services provided by forests and bogs, which manifests in an increased focus on health and well-being, cultural heritage values, recreational opportunities, and also economically significant activities [1]. Despite the urbanization processes, the traditions of collecting berries and mushrooms are persistent in the Nordic and Baltic countries. Even currently, wild berries—such as cranberries, blueberries and lingonberries—are collected in economically significant quantities both for self-consumption and as a highly demanded market product. Picking of wild cloudberry *Rubus chamaemorus* L. (*Rosaceae*) is also a popular local activity, mainly in northern Europe, where—in suitable conditions—wild stands can provide abundant harvests [2,3]. In Latvia, although less commonly found, the cloudberry still sustains large stands and provides a good harvest of these tasty and healthy berries.

Since the 1960s, the distribution and growth characteristics of cloudberry in the main growing regions of Scandinavia and Canada and its cultivation potential have been well described [3,4,5,6,7,8]. However, in the context of climate change and the decline of suitable habitats over the last decades, there is a lack of recent studies on changes in the distribution of cloudberry worldwide. There are very few data on the cloudberry population in the Baltic states, including Latvia, which would characterize the growth habitat, surrounding vegetation structure, the agrochemical parameters of the soil, and the nutrient status of the species.

Cloudberry is a dioecious perennial plant with a boreal circumpolar distribution, occurring naturally throughout the Northern Hemisphere [4]. The species inhabits mainly sphagnum bogs, marshes, tundra, and boreal forest habitats that are generally wet, acidic, and poor in nutrients [2,9].

*Rubus chamaemorus* is a herbaceous plant with small vegetative parts (5-25 cm) and with long (up to 10 m) creeping rhizomes below the soil surface. Leaves are rounded with five or seven lobes and have average length/width of 3–6 cm. In Latvia, cloudberries bloom in May and early June, and fruits start to appear and ripen in August. Unripe fruits are red but, ripened fruits turn soft orange [2,10].

The main form of reproduction is vegetatively by clonal growth, and since *R. chamaemorus* is dioecious, only female plants produce fruit [3]. Wild cloudberry stands usually have a high male–female ratio, and male flowers are larger, which affects pollination and fruit yield [11,12].

The southernmost distribution limit of the cloudberry coincides with the northernmost regions of Germany, where it is extremely rare, and Poland, where the cloudberry is a specially protected glacial relict species found in nature reserves and listed in the *Polish Plant Red Data Book* [13,14]. Rare occurrences have also been found further south in mountain ranges both in Poland and the Czech Republic, where it grows in subalpine marshes [14,15]. Their general absence is marked in wet areas with long-lasting and continuous rainfall, such as the Scottish islands [5]. In Latvia, cloudberry is not recognized as endangered and does not have protected species status. However, the distribution and vitality of currently existing cloudberry stands can be negatively affected by factors such as deforestation, bog extraction for horticultural peat harvesting, and climate warming.

Based on the dendroflora database of Latvia [16], cloudberry is distributed mainly in the central and northern parts of the country, which coincides with the prevalence of peat bogs and climatic conditions suitable for cloudberry. Significantly fewer or no records are found in the far eastern regions of Latvia, although they are regionally rich in bogs. This also coincides with more extreme seasonal fluctuations in the eastern continental region as compared to coastal and central regions where the climate is milder.

In the Northern regions, as well as in Latvia, cloudberry is found mainly in sphagnum moss bogs and bog pinewoods; it grows less frequently in open areas in bogs and more often in bog–woodland transition zones, where sphagnum patches are abundant [2,10].

Climatic conditions in Latvia are suitable for both the producing and overwintering of cloudberries, and it would be important in the future to model how climate change in different areas will affect the species that produce some of the most valuable berries in the northern region. Studies have shown that low-to-moderate mean temperatures are important not only for the growth of cloudberries but also for the quality of the fruits, especially during ripening, although frosts during flowering are a fruit-reducing factor [3,17].

In Latvia, wild cloudberry is considered an expensive delicacy. The average retail price of cloudberry in 2017 and 2018 reached EUR 7.00 per L [18]. This is determined by both the excellent taste and aroma characteristics and the high content of health-promoting compounds. Both cloudberry fruits and leaves have been shown to have a unique composition of bioactive compounds, especially ellagitannins, with antimicrobial, antioxidant, and anticancer properties [19,20]. The relatively low availability of these berries on the market in Latvia is an important factor as well. Studies conducted in different countries have shown that in natural habitats, cloudberries have a low and variable yield, with average fruit yield in productive peatlands reaching 300 kg ha^−1^ [3]. The berry yield is limited by several factors—mainly climate conditions, pollination success, unfavourable proportion of female and male plants, specifics of cloudberry reproduction and associated resource limitations, and nutrient availability [3,21,22].

Taking into account the economic and ecological importance of possible cloudberry commercial cultivation in Latvia, research on this issue has aroused wide interest. Currently, the areas of active peat extraction sites in Latvia comprise 25,000 ha, and the after-use of extracted peatlands after their abandonment is a topical issue [23]. Given the large areas of extracted peat bogs where the residue peat layer is sufficiently thick, the cultivation of cloudberry in Latvia could be a promising sector which would at the same time relieve the impacts on natural cloudberry stands. In this context, complex investigations of wild cloudberry in natural populations are especially important to clarifying the growth habitat conditions and potentially promoting the vitality of cloudberry stands. The aim of the present research was to explore wild cloudberry growth conditions in Latvia, including habitat characteristics, the composition of surrounding vegetation communities, and the peculiarities of plant mineral nutrition. The knowledge gained in this study will be a valuable basis for the development of cloudberry cultivation technologies in the hemiboreal climate zone.

## 2. Results

### 2.1. Growing Conditions and Surrounding Vegetation Composition

Most of the surveyed areas were forested pine and birch bog habitats, with some exceptions—scarcely forested peatlands or bog–forest transition zones. Thus, shading conditions also varied between sites; shade was provided mostly by trees of various heights as well as shrubs of various species.

In wooded areas where cloudberry grows, the accompanying tree species were mainly conifers, dominated by Scot’s pine (*Pinus sylvestris)*, often interspersed with birch (*Betula pendula)* and—in some places with Norway—spruce (*Picea abies)*. In bog areas, the main tree species were *Betula pendula* and *Pinus sylvestris* saplings.

Based on the vegetation records, we found that cloudberry populations were associated with 31 species in total (Table 1).

The highest number of species in the cloudberry study area was found in the Melnais Lake Mire site—19 species. This was followed by the Cenas Moorland area (17 species) and Raganu Bog (Kemeri National Park area) (15 species). The lowest species diversities were found in Lauga Bog (5 species), Dzelve Bog (6 species), and the territory of the Great Kangari Lake (7 species).

Herbaceous vegetation was the most diverse in terms of species and on average occupied 45% of the sample plot area (20 species in total). The bryophyte/lichen layer was represented by 12 species and on average occupied 40% of the area. The tree layer was not represented by any species in any of the plots, although tree species were present. These were mostly young trees or saplings (under 10 cm) and represented either the shrub or herbaceous layers. The shrub layer (3 species) was represented by trees—*Betula pendula* Roth, *Picea abies* L., and *Pinus sylvestris* L.

**Figure 1:**
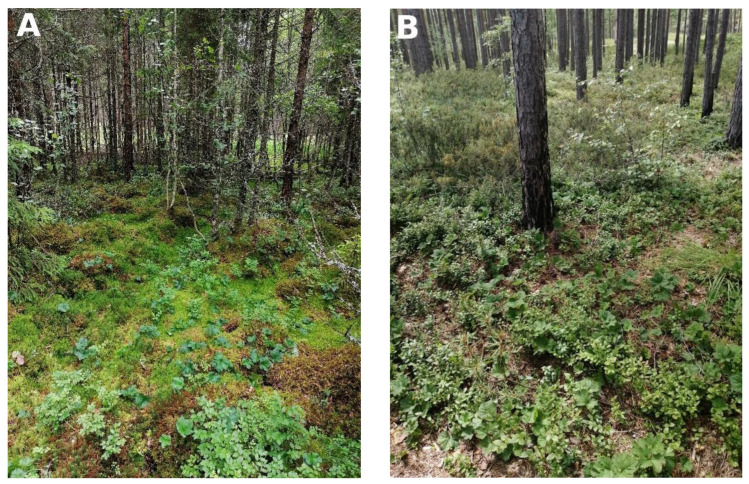
Habitats of wild cloudberry representing two plant community classes, (**A**) Cl. *Oxycocco-Sphagnetea* and (**B**) Cl. *Vaccinio-Piceetea* (photos by L. Āboliņa).

In general, the species most frequently found with the cloudberry were Magellan’s sphagnum (*Sphagnum magellanicum* Brid.), bilberry (*Vaccinium myrtillum* L.), and bog cranberry (*Oxycoccus palustris* L.). Based on Latvian forest typology [24] and a list of plant communities in Latvia developed by Laiviņš [25,26], sites corresponded to two main plant society classes—*Oxycocco-Sphagnetea* and *Vaccinio-Piceetea* (Figure 1). Class *Vaccinio-Piceetea* was represented by associations of pine forest plant communities *Vaccinio myrtilli-Pinetum* (sites no. 5, 11, 18), *Vaccinio vitis-idaeo-Pinetum* (site no. 3)*,* and *Vaccinio uliginosi-Pinetum* (site no. 6). Class *Oxycocco-Sphagnetea* was represented by association *Sphagnion magellanici* (sites no. 2, 10, 12, 13, 17)*,* where the dominating species were sphagnum mosses as well as *Andromeda polifolia* and *Oxycoccus palustris.*

**Figure 2:**
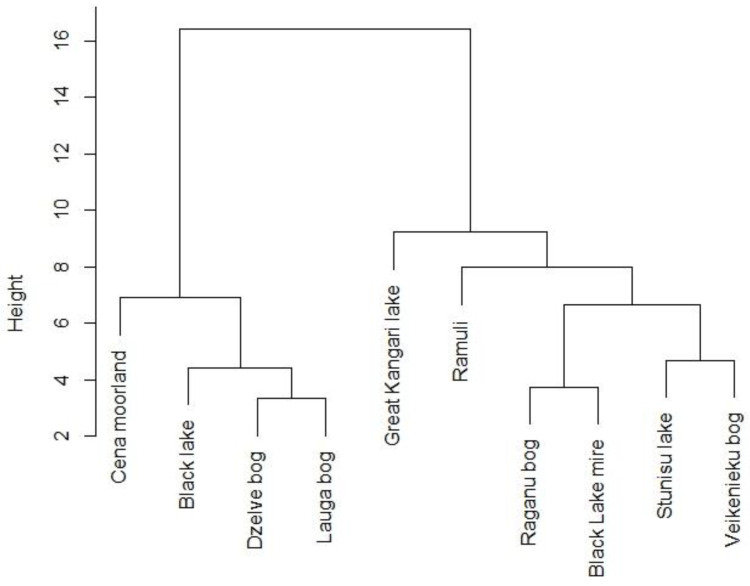
Dendrogram of hierarchical cluster analysis of cloudberry study sites based on vegetation composition. Levels of similarity between different sites are indicated by vertical linkage distance.

Hierarchical clustering of vegetation data was performed to analyze similarities in species composition and their potential relationship with factors such as plant community type, geographic location of study sites, and proximity to peat extraction sites. *Results* revealed, which cloudberry study sites were closest in terms of species composition (Figure 2). Three pairs in two main groups of sample sites were found to be closest to each other (Dzelves–Laugas Bogs, Raganu—Melnezers Bogs, Stunisu–Veikenieku Bogs) with the highest similarities in species composition.

Dzelve and Lauga Bogs are geographically close to each other and were also found to be the poorest in terms of species; the herbaceous vegetation consists mainly of the genus *Vaccinium* and the moss vegetation of one species *Pleurozium schreberi* (Brid.) Mitt. Meanwhile, Cena Moorland, Great Kangari Lake, and Ramuli differed the most, as they placed farthest above the rest of the sites.

In general, hierarchical clustering of vegetation data revealed that similarities in species composition did not correspond to other site characteristics regarding either plant community types or geographic location.

### 2.2. Mineral Nutrition of Wild Cloudberries

**Figure 3:**
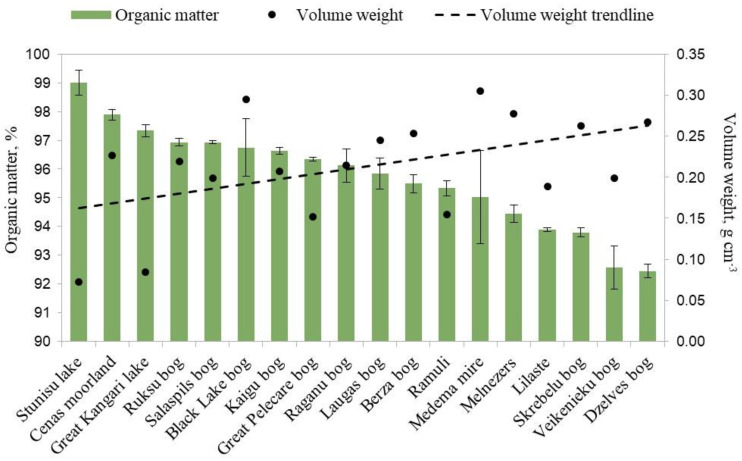
Organic matter content (%) and volume weight (g cm^−3^) of peat in wild cloudberry study sites in Latvia, 2020–2022.

All of the soil samples had high contents of organic matter in the range of 92.45–99.01%. It is generally accepted that soil containing more than 75% plant organic matter is classified as peat [27]. In general, the contents of organic matter (%) were similar between almost all of the study sites (Figure 3). Peat with the highest organic matter content was detected in site Stunisu Lake, characterized by almost completely intact sphagnum moss in which cloudberry rhizomes were found. The lowest content of organic matter in the growing medium was found at the Veikenieki and Dzelve Bogs research sites. The volume weight range was within 0.07–0.30 g cm^−3^.

The degree of peat decomposition for most of the sites was evaluated as slightly decomposed peat (H1–H3 on the von Post humification scale). The von Post values for Skrebelu, Veikenieku and Dzelves bogs were slightly higher (H4–H5), indicating more decomposed peat in sites close to current peat extraction areas.

The pooled sample data revealed a negative correlation between organic matter content and volume weight (*n* = 18, *p* < 0.05, *r* = −0.469), confirming that higher levels of peat decomposition increase peat density but decrease organic matter content. Therefore, in the study sites in Latvia, cloudberries generally grew in peat with very low mineral content and a low degree of decomposition.

Overall, the chemical composition analysis of the peat samples revealed a wide range of nutrient concentrations (Table 2). Among the macronutrients, the widest range was found for N and Ca, whose concentrations differed by 23 and 17 times, respectively, when comparing the minimum and maximum values. Of the microelements, the highest ratio of maximum and minimum values was found for Mn and Fe—29 and 90 times, respectively. Thus, the coefficient of variation (CV) was also rather high for most of the nutrients, especially for S (71.58%) and Mn (129.89%). The smallest differences between sites were found in soil reaction (pH) indicators (CV 14.48%), thus confirming that all of the sites were represented by acidic peat: mean pH range was within 2.68–3.32 (Kaigu Bog and Great Kangari Lake, respectively).

The average Ca:Mg ratio in the soils of the study sites was 4.06 ± 0.32, ranging from 0.69 (Kaigu Bog) to 7.71 (Raganu Bog), thus indicating on the possible impact of different soil bedrock in different locations.

**Figure 4:**
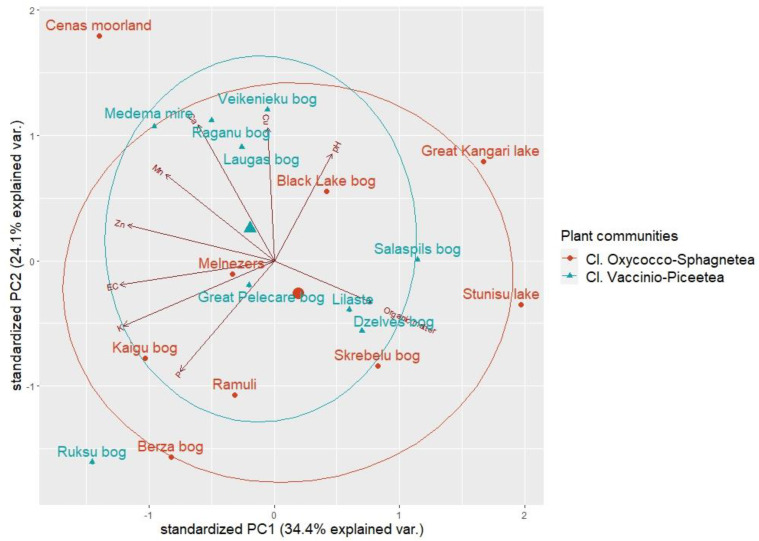
Distribution of the cloudberry study sites within the axes of principal component analysis (PCA) of the soil chemical dataset. Larger shapes indicate centroids of groups.

The PCA of the results of peat analysis revealed three principal components (eigenvalue > 1.0) that explained 70.7% of the total variance (Figure 4).

The most important factors in the principal component analysis of peat chemical composition were organic matter content, soil acidity, and nutrient (Ca, Cu, K, Zn) levels. The first principal component (PC1) had positive associations with the organic matter content and pH levels of the peat samples but negative correlations with nutrients. However, based on eigenvector values, organic matter content and pH levels had the strongest correlations with PC3 (Table 3). PC2 had strongest positive correlations with the nutrient component.

Results indicate that PC3 correlates with peat structural parameters, such as decomposition and acidity, but that PC2 correlates with nutrient concentrations of the peat. PC1 in this case corresponds to how the decomposition of peat (decrease of organic matter) increases the nutrient concentrations and volume weight of the peat.

Because individual sites were scattered in the ordination space, the PCA results also confirmed that study sites did not differ when grouped by plant communities, as indicated by group centroid proximity.

The concentration of nutrients was determined also in cloudberry leaf samples (Table 4). In general, the results revealed lower heterogeneity, especially in leaf macronutrient concentrations in comparison to soil chemical results. However, several substantial differences were found: for macronutrients, the concentration of Ca varied 6.0 times when comparing the maximum and minimum values; for micronutrients, the highest max/min concentration ratio was found for Mn (13.0 times), Mo (7.5 times), and Cu (6.9 times).

When analyzing nutrient uptake in the soil–plant system, there were few statistically significant (*p* < 0.05) correlations, none of which were close (Table 5). Negative correlations found between soil parameters and nutrient content in cloudberry leaves were between N and Zn; Mg and K; Cu and Ca, Zn, and B; Zn and S; and Mn and K (−0.27 < *r* < −0.37). Positive relationships were found between Mn content in peat and leaves and Mo in peat and leaves (*r* = 0.31, *p* < 0.05). Statistical analysis showed that Mn concentrations in cloudberry leaves were significantly related to P, K, and Fe supply in the peat. A similar relationship was also found for Zn with P and for P with S (*r* > 0.27, *p* < 0.05).

## 3. Discussion

### 3.1. Habitat Characteristics

Considering cloudberry is usually described as a species mainly found in oligotrophic sphagnum bogs (also called raised moss peat bogs), the results of our study confirmed sphagnum as the most frequently found companion species for cloudberry stands. This factor is also taken into account when cultivating cloudberry in cutover peat bogs in Scandinavia and North America. It is recommended to plant sphagnum moss before planting cloudberry to improve peat structure and provide poorly decomposed peat that is most suitable for cloudberry cultivation [31].

The fact that herb and bryophyte layers were the most represented in the cloudberry study sites compared to the shrub layer could indicate that cloudberry avoids low-ground-shade habitats. Shading on this level is provided by nearby shrubs, such as different *Vaccinium* species, *Ledum palustre,* or *Calluna vulgaris.* Shading from older trees, especially pines and birches, which are the main tree species in these habitats, seems to provide necessary sunlight conditions, as cloudberry is mostly found in partly-to-fully forested bog areas. Our study suggests that *Rubus chamaemorus* is likely to be a species that prefers medium levels of direct sunlight (not full light, but not full understory shade either). Further research on optimum lighting conditions is needed.

In general, the species composition of cloudberry study sites in Latvia corresponded to two plant community classes: Cl. *Vaccinio-Piceetea* and Cl. *Oxycocco-Sphagnetea. Vaccinio-Piceetea* is a typical plant society of boreal coniferous forests, particularly pine and pine–birch wetlands in Latvia [32]. Associations of *Oxycocco-Sphagnetea* dominate in raised moss peat bogs, including forested bogs [33]. As results suggest, these habitats, as well as transition zones between them, are most suitable habitats for cloudberry growth in the climate of Latvia.

The choice of research sites in the main distribution area of cloudberry in Latvia and the results of inspection have proven that cloudberry prefers peatlands in both natural and previously managed or disturbed places. Species found growing together with cloudberry are typical Northern hemisphere species (Genus *Vaccinium* and *Sphagnum*). Taking into account the similar requirements of these species for acidic and highly organic soil (peat) and the relatively low demand for nutrients, their presence is considered a good indicator that the area could be suitable also for cloudberry. As Latvia is rich in forests, bogs, and extracted peatlands [23,34], there are currently large areas of suitable habitats.

Although the climate in Latvia is appropriate, the country is situated close to the southern boundary of the distribution of cloudberry stands. *R. chamaemorus* is already very rare in nearby countries south of Latvia, such as Poland and Belarus, where it is considered an endangered species and is legally protected by the state [13,35]. Due to climate change, boreal forests and peatlands are predicted to experience dramatic alterations, especially in the hemiboreal zone [36,37]. Changes in peatland vegetation phenology and composition will have complex consequences. Species that are unable to adapt to the changed climatic conditions are in great danger of extinction. In this aspect, long-term studies on the changes in distribution, vitality, and abundance of the cloudberry population in Latvia under climate change would be necessary.

Because cloudberry most often grows in peat, it is also often found in close proximity to commercial peat extraction sites (in this study, 5 out of 18 sites were close to abandoned or functioning peat extraction sites). It has been proven that a low degree of peat decomposition is one of the main factors that also determines the success of cloudberry plantations [31]. In Latvia, less decomposed and acidic sphagnum peat is the main type of peat being mined for the production of horticultural substrates [38]. Although some territories may have been lost to peat mining sites, there is currently no evidence to suggest peat extraction is a threat to cloudberry populations.

According to the Right to Roam, people are allowed free movement in all state, municipality and private forests, unless there are prohibition signs, and it is permitted to gather berries for private consumption and sale, except in strongly protected nature areas [39]. However, the low and variable yield of cloudberry in natural habitats should be taken into account.

### 3.2. Mineral Nutrition Status

Since cloudberry is a highly demanded and valuable berry and could be considered a potential species for the recultivation of abandoned extracted bogs, it is necessary to develop both growing technologies and fertilization systems for cloudberry cultivation. *R. chamaemorus* is generally considered to be a species that prefer nutrient-poor and acidic environment—peat soils. However, there is very little information concerning the exact mineral composition of cloudberry leaves [40,41,42] and plant-available concentrations of nutrients in peat, especially for the hemiboreal climate zone. For starting plant introduction or cultivation, species-specific adequate soil properties and the surrounding microclimate are critical parameters that must be ascertained and evaluated.

In general, low concentrations of plant-available N, P, and S in peat were found in most of the cloudberry study sites. Our results revealed that overall peat nutrient concentrations for cloudberry were slightly higher than previously determined for wild cranberry (*Vaccinium oxycoccus* L.) [43] or comparable to the levels for wild blueberry (*Vaccinium myrtillus* L.) [44] in Latvia. It should be noted that the levels of nutrients found in peat were significantly lower than recommended for cultivated berry crops that prefer acidic environments, such as American cranberries (*Vaccinium macrocarpon* Aiton) and highbush blueberries (*Vaccinium corymbosum* L.) [29,30].

As cloudberry sites in Latvia are found on peat soils, an additional influx of N, P, and S could occur in summer as a result of the mineralization and nitrification processes of organic matter. It has been established that cloudberries are able to absorb both forms of mineral nitrogen available to plants—NO_3_^−^ and NH_4_^+^—but the form of NH_4_^+^ is absorbed more efficiently, which is also explained by the low availability of NO_3_^-^ in peat soils. There is also evidence for the ability of cloudberry to uptake N in organic forms under natural conditions [45].

The smallest variation in leaf nutrient concentrations between sites corresponded to N, thus indicating how cloudberry could successfully accumulate N at the required level regardless of N availability in the soil. This study showed N contents of the wild cloudberry leaves in the range of 1.9–3.0%, corresponding to the sufficiency range reported for highbush blueberry, strawberries (*Fragaria x ananassa* Duch.), and raspberries (*Rubus idaeus* L.) [29,46,47,48]. Nitrogen fertilization would likely maintain/increase foliar N content, thereby providing optimal conditions for both rhizome and root growth as well as for intensive vegetative growth and berry production in commercial plantations.

Previous studies in Canada have indicated that total leaf area and leaf N concentration were the most important factors explaining root system development for cloudberry rhizomes transplanted in cutover peatland [49]. This is mainly explained by more intense photosynthesis and higher carbon fixation.

While no significant differences in N uptake between wild and cultivated plants within the same species have been found, overabundance of other nutrients (such as K and P) can be toxic to wild plants, while cultivars can adapt to a wider range of contents [45]. This suggests that the level of K and P in cloudberry cultivars could be higher than in the wild, especially if fertilization is applied, but the fertilization should be very precise and not excessive.

The mean leaf P, K, and S concentrations in wild cloudberries in Latvia reached 0.15%, 1.16%, and 0.14%, respectively. These concentrations were in the range reported from natural sites in Scotland, North Wales, and Canada [40,42,50] and marginally confirmed the minimum sufficiency requirements established for strawberries and raspberries—0.2% for P, 1.3% for K, 0.15% for S [46]—but fully met the requirement for blueberries—0.1% for P and 0.35% for K, 0.1% for S [47,48].

In natural peat bogs, cloudberries produce only a few roots along the rhizomes but can develop a large root system when grown in a fertilized substrate in containers [31]. Fertilization, especially optimal P supply, stimulates root growth in many species [51,52]. Leaf N/P mass ratio is one indicator of nutrient limitation in the growth of wild species [51]. In nutrient-poor environments, such as cutover peatlands, P can be a limiting factor for the establishment of cloudberries. Previous studies on natural sites in Europe have reported slight-to-intermediate N limitation in cloudberry based on leaf N/P ratios (N/P 7–14) [41]. In Canada, colimitation of N and P was found [50]. The average N/P ratio of cloudberry leaves in Latvia (N/P 17.6 ± 0.7) exceeded the limit of the ratio N/P ≥ 16 [53], which indicates P as the limiting nutrient. However, it is likely that cloudberry growth could be limited by several nutrients, including N, P, and S. Since rhizome growth and root formation are primarily driven by carbohydrate availability, complex fertilization most likely is necessary to enhance cloudberry vegetative growth and photosynthesis. The need for a complex approach is also confirmed by the relatively close positive correlation between N, P and K in cloudberry leaves (0.47 > *r* < 0.57, *p* < 0.05).

Since the 1990s, atmospheric sulfur dioxide emissions and thus delivery to forest ecosystems have been significantly reduced worldwide [54]. Consequently, plant S status is highly dependent on plant-available S in the soil. As the peat soils from the cloudberry sites in Latvia were not rich in S, and intensive mineralization of poorly decomposed peat is not expected, the use of sulfur-containing fertilizers could be considered for the successful cultivation of cloudberries in extracted bogs.

The contents of Mg found in wild cloudberry leaves could be considered as high for wild plants—on average 0.52% Mg—and were close or even higher than those of Ca (0.50%). Ca and Mg composition determined for cloudberry stands in Canada (Quebec and Labrador bogs) also revealed similar foliar ranges of these nutrients: Mg 0.37–0.86%, Ca 0.20–0.88% [42]. In other berry shrubs grown in peat—for example, both wild and cultivated cranberries and blueberries—Ca levels in leaves are usually 2–3 times higher than Mg [55]. It is possible that this is typical for berries of the genus *Rubus*, as the standard range for Mg in raspberry and blackberry (*Rubus allegheniensis)* leaves is 0.6–0.9%, and that of Ca is 0.60–2.0% [28,46,56,57]. However, the difference in the ranges of these nutrients, which is greater for Ca, indicates a possible need for additional Ca supply to ensure successful growth and berry quality of cultivated cloudberry.

The content of not only macronutrients but also micronutrients in the soil and their availability and uptake could be of great importance in ensuring the growth, development, and berry production of cloudberry. Fe, Mn, and Zn concentrations in peat soils can be characterized as not limiting for cloudberry, as they corresponded to the levels recommended for cultivating *Vaccinium* berry species [29,30] in extracted peat bogs. Contrarily, the contents of Cu, B, and Mo were assessed as low and possibly limiting to population vitality and productivity.

In the given soil conditions, a generally high micronutrient status was found in the leaves of wild cloudberry in Latvia. Indeed, the mean concentrations of Fe, Mn, Zn, and Cu corresponded to optimal levels for most cultivated fruits and berries reported by different authors [46,48,56]. This can be explained by (a) the acidic reaction of the soil, which is considered one of the main factors contributing to the absorption of micronutrients [51], (b) by sufficient nutrient content in soil (in the case of Fe, Mn, Zn), and (c) by high uptake capacity of cloudberries to accumulate microelements even in low soil supply (in the case of Cu and B) conditions.

In general, mean cloudberry leaf B was slightly below the optimal range of 20–70 mg kg^−1^ proposed by Hart et al. [47] and Pritts et al. [46] for blueberries, cranberries, strawberries, and raspberries.

Mo is the only micronutrient with lower availability in acid soils [51]. Therefore, it is not surprising that under conditions of low Mo concentration in peat, the mean Mo concentration found in the cloudberry leaves was also low (0.35 mg kg^−1^) and below 0.5 mg kg^−1^, which was proposed by Nollendorfs [29,30] as the sufficiency threshold for *V. corymbosum* and *V. macrocarpon*. The plant-available B (BO_3_^−3^) and Mo (MoO_4_^−^) in the soil are in the anion forms and thus highly leaching from light acid soils, especially in conditions of high rainfall and water level fluctuations. As B is essential for root growth, reproductive organ development, and pollination success, and Mo for N metabolism in plant and pollen viability [51], their adequate supply is necessary to ensure a good fruit set. Since a significant correlation was found between the Mo content of peat and cloudberry leaves, the inclusion of Mo in the composition of fertilizers could be recommended. Considering the narrow range between boron toxicity and deficiency in the soil [58] and the high potential for leaching, foliar fertilizers are likely to be more suitable for providing optimal B nutrition.

In general, high nutrient uptake capacity was found for wild cloudberry growing in a nutrient-poor environment, as most of the leaf nutrients corresponded to the sufficiency levels determined for different cultivated berries. An optimal supply of nutrients significantly enhances the process of photosynthesis and ensures the subsequent transport of photoassimilates over long distances [51]. It is especially important for cloudberry, a species with specific morphology—approximately 95% of its biomass is located underground, only 5% is above ground, and each shoot has only one to three leaves [59]. Therefore, to avoid mineral nutrition becoming the limiting factor in berry production for cultivated cloudberries, leaf testing would be highly recommended to obtain information on the actual nutrient status of plants.

## 4. Materials and Methods

### 4.1. Inspection in Natural Habitats

**Figure 5:**
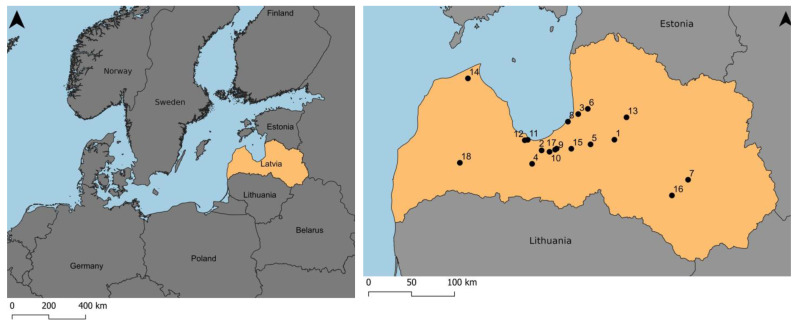
Location of cloudberry study area in Latvia. Study sites: 1—Berzu Bog, 2—Cena Moorland, 3—Dzelves Bog, 4—Kaigu Bog, 5—Great Kangari Lake, 6—Lauga Bog, 7—Great Pelecare Bog, 8—Lilaste, 9—Medema Bog, 10—Black Lake Mire (Olaine Parish), 11—Melnezers (Jurmala State City), 12—Raganu Bog, 13—Ramuli, 14—Ruksi Bog, 15—Salaspils (Zeltini) Bog, 16—Skrebeli Bog, 17—Stunisu Lake, 18—Veikenieki Bog. Five sites are near to currently active or abandoned peat mining sites (sites no. 3, 4, 9, 16, 18); 13 of the sites are natural and/or recreational areas (sites no. 1, 2, 5, 6, 7, 8, 10, 11, 12, 13, 14, 15, 17). Phytosociological records were taken in sites no. 2, 3, 5, 6, 10, 11, 12, 13, 17, 18.

Based on the information about the existing viable populations of cloudberry in Latvia [16], 18 stands were surveyed in the vegetation seasons (June–August) from 2020 to 2022 (Figure 5).

**Figure 6:**
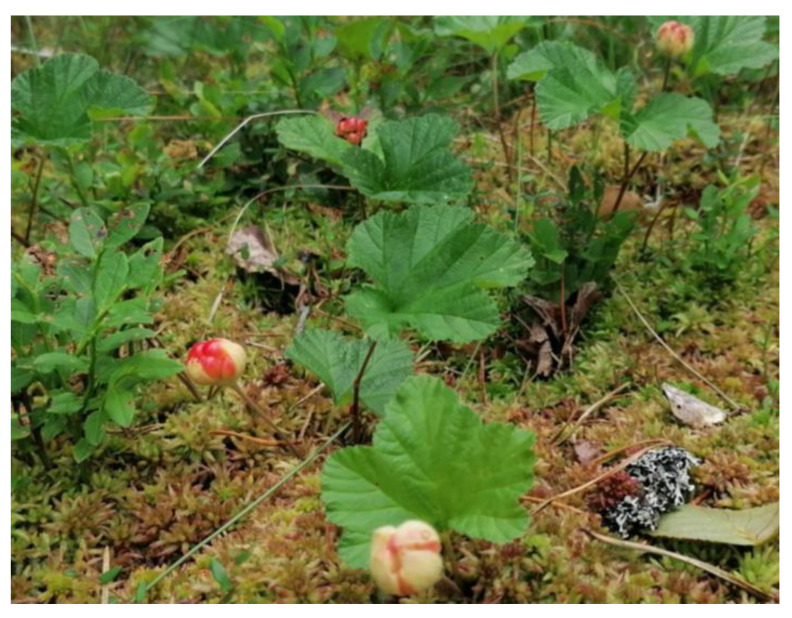
Wild cloudberry with visibly red (unripe) fruits (photo by L. Āboliņa, 6 July 2020, Latvia).

Samples were collected throughout the growing season before, during, and after the flowering/fruiting period. As the timing of these periods vary from year to year, the date of taking samples is provided (Table 6). In flowering/fruiting season both flowers and fruits in various stages of development were observed in the cloudberry stands (Figure 6).

The study sites, with a few exceptions, were chosen in the main distribution area of cloudberry in Latvia—in central and northern regions—which is located in the hemiboreal climatic zone. The climate in Latvia is moderately warm and humid: the average annual precipitation is 685.6 mm, and the average temperature is −3.1 °C in February, but +17.8 °C in July (records from 1991 to 2021, State Ltd Latvian Environment, Geology and Meteorology Centre) [60]. The average annual temperatures of the coastal region are up to 2 °C higher than those of the eastern continental region, where seasonal temperature fluctuations are more intense.

A description of the surrounding vegetation was carried out for 10 study sites, listing the most common companion species that occur together with cloudberry. General growing conditions, such as biotope and shading, were also assessed in all sites. Soil and leaf samples were collected for agrochemical analyses in all study sites to assess the nutrient status of the wild cloudberry in Latvia.

### 4.2. Phytosociological Records

In order to determine which species are most frequently found together with cloudberries, relevés (lists of species and vegetation cover in a plot) were used. For each site, five 1 × 1 m plots were randomly selected, and the coverage of all growing tree, herbaceous, and bryophyte/lichen species found was recorded in prepared forms using the Braun–Blanquet scale [61], and the percentage cover of each layer was estimated. For each plot, coordinates and photographs were taken.

### 4.3. Nutrient Analysis

Cloudberry leaf samples were collected at each study site by walking around the area in a radius of at least 100 m and picking randomly selected leaves, resulting in three samples of at least 50 leaves each. In all inspections (Table 6), the most recently matured leaves were collected. The collected samples were quickly washed with distilled water, dried at +60 °C, finely ground using a laboratory mill, then dry-ashed in concentrated HNO_3_ vapors and redissolved in 3% HCl for K P, Ca, Mg, Fe, Cu, Zn, Mn, B, and Mo detection. Wet digestion was used for N (in H_2_SO_4_) and S (in HNO_3_) determination in plant samples.

From each study site, three soil samples were taken from the plant root zone at depths of 0 to 20 cm. Each sample consisted of five thoroughly mixed subsamples. Soil was air-dried and sieved through a 2 mm sieve. To determine plant-available nutrient (N, P, K, Ca, Mg, S, Fe, Mn, Zn, Cu, Mo, B) concentrations, soil samples were extracted using 1 M HCl in volume ratio 1:5 as previously described [62]. The obtained results of nutrients in soil were expressed in mg L^−1^ because the growth of plant roots take place in a certain volume, and in peat soil, this volume differs substantially from the weight of this volume. Nutrient content in mg kg^−1^ may give a false impression of a high level of nutrient supply in peat soils with low bulk density as only a small proportion will be available to the plant.

For both plant and soil samples, the levels of Ca, Mg, K, Fe, Cu, Zn, and Mn were estimated using microwave plasma-atomic emission spectrometer (AES (MP-AES) 4210 Agilent Technologies) [63]. The contents of P, Mo, N, S, and B were determined via colorimetry: P—using ammonium molybdate in an acid reduced medium, Mo—using thiocyanate in reduced acid medium, N—using Nesler’s reagent in an alkaline medium (modified Kjeldal method), B—using hinalizarine in sulphuric acid medium, S—using the turbidimetric method by adding BaCl_2_. The concentrations of all nutrients for soil samples are given as mg L^−1^. All spectrometric, colorimetric, or photometric measures were performed in triplicate.

Soil electrical conductivity (EC) was measured in soil via distilled water extraction (1:5) with the Hanna Instruments 215 EC conductivity meter. Soil reaction (pH) was measured in 1 M KCl soil–extractant mixture (1:2.5) using the pH meter Basic Meter PB-20.

Soil (peat) organic matter content (%) was determined via the loss ignition method by combustion at 450 °C; volume weight was estimated by weighting the dry mass of the peat (after heating at 105 °C for 24 h) per the known volume. The degree of peat decomposition was evaluated using the von Post scale [64].

### 4.4. Statistical Analysis

Statistical analysis was performed using the R programming language—the base package and the collection of packages “Tidyverse” provided both statistical and graphical data science tools [65,66].

Mineral nutrition data were analyzed with descriptive statistics. The coefficient of variance (CV) in percent was calculated to characterize the heterogeneity of nutrient concentrations. Hierarchical clustering (method: Ward, distance: Euclidean) was performed for species composition and mineral nutrition data to find similar sites. Principal component analysis (PCA) was performed for mineral nutrition data, grouping study sites by main plant communities.

## 5. Conclusions

A complex study was carried out to provide insight into the growth conditions of wild cloudberry in Latvia, located in the hemiboreal climatic zone. With this work, we have taken an additional step to better understand the principles of choosing planting sites depending on the soil characteristics, ensuring appropriate light conditions and an optimal mineral nutrition regime for the initiation of cloudberry cultivation in Latvia. The results clearly indicated acid peat soils with high organic matter content and low degree of decomposition as the proper sites for cloudberry cultivation. Our study suggests that *Rubus chamaemorus* is likely to be a species that prefers medium levels of direct sunlight, and ensuring optimal light conditions could be a challenge for commercial cultivation. However, further research is needed in this direction. In general, high nutrient uptake capacity was found in wild cloudberry growing in nutrient-poor environments, as most leaf nutrients corresponded to the optimal levels determined for different cultivated berries. However, balanced fertilization to ensure good vegetative and root growth of the plant would be recommended. Although the first results on wild cloudberry in Latvia indicated that optimization of P, S, B, and Mo should be the main focus, further research in this area is needed. Additionally, studies are necessary to understand the factors that affect berry yield formation and size.

## Figures and Tables

**Table 1:** Vegetation composition according to Braun-Blanquet scale and vegetation cover of layers (%) in cloudberry study sites in Latvia, 2020–2022.

Cover	Study Site									
	Cena Moorland	Dzelve bog	Great Kangari Lake	Lauga bog	Black Lake mire	Melnezers	Raganu bog	Ramuli	Stunisu Lake	Veiukenieku bog
Tree layer, %	-	-	-	-	-	-	-	-	-	-
Shrub layer, %	5	-	-	-	2	1	2	20	10	15
Herb layer, %	50	50	65	30	65	60	40	25	55	25
Bryophytes/lichen layer, %	20	60	30	25	35	30	55	60	25	65
Without vegetation, %	35	-	5	55	5	30	10	-	15	5
Shrubs										
*Betula pubescens*	-	-	-	-	-	-	-	-	-	1
*Pinus sylvestris*	-	-	-	-	-	1	1	-	-	-
Herbaceous										
*Andromeda polifolia*	1	-	3	-	4	-	5	-	5	3
*Betula pubescens*	-	-	-	-	2	-	1	-	-	2
*Calluna vulgaris*	5	-	-	-	5	-	5	-	2	4
*Carex lasiocarpa*	-	-	-	-	2	1	2	5	4	5
*Carex nigra*	-	-	-	-	1	-	-	-	-	2
*Drosera anglica*	-	-	-	-	-	-	-	-	2	-
*Empetrum nigrum*	1	-	2	-	-	-	-	-	-	-
*Ledum palustre*	3	5	-	-	2	2	1	-	3	5
*Lycopodium annotinum*	-	-	-	-	-	-	-	-	-	2
*Melampyrum pratense*	2	-	2	-	-	3	-	-	-	4
*Oxycoccus microcarpa*	-	-	5	-	-	-	-	-	-	-
*Oxycoccus palustris*	4	-	-	-	4	-	4	-	5	2
*Picea abies*	-	-	-	-	-	-	1	1	-	2
*Pinus sylvestris*	-	-	-	-	3	1	-	-	3	-
*Rubus chamaemorus*	2	1	1	1	2	1	2	3	1	2
*Uzula pilosa*	-	-	-	-	-	1	-	-	-	-
*Vaccinium myrtillus*	4	2	-	2	-	5	-	3	5	1
*Vaccinium uliginosum*	-	2	-	3	-	-	3	2	2	1
*Vaccinium vitis-idaea*	1	5	-	2	-	3	-	-	-	-
Bryophytes/lichens										
*Aulacomnium palustre*	-	-	-	-	1	-	2	-	-	-
*Cladonia sp.*	1	-	-	-	1	-	2	-	-	-
*Dicranum bonjeanii*	3	-	-	-	1	-	-	-	-	-
*Dicranum polysetum*	-	-	2	-	-	1	-	-	-	4
*Dicranum scoparium*	4	-	-	-	1	5	-	-	-	-
*Hylocomium splendens*	1	-	-	-	-	2	-	-	-	-
*Pleurozium schreberi*	4	5	-	5	-	3	-	3	-	-
*Polytrichum commune*	-	-	-	-	-	-	-	3	-	-
*Polytrichum strictum*	-	-	-	-	1	-	4	-	-	-
*Rhytidiadelphus triquetrus*	-	-	-	-	-	-	-	-	2	-
*Sphagnum angustifolium*	-	-	-	-	2	-	4	3	-	1
*Sphagnum capillifolium*	-	-	-	-	1	-	3	-	-	-
*Sphagnum magellanicum*	-	-	5	-	3	-	5	5	3	5

**Table 2:** Nutrient concentration (mg L^−1^, 1M HCl extraction), peat pH and electrical conductivity (EC, mS cm^−1^), and organic matter content in air-dried peat samples (0–20 cm) from cloudberry study sites in Latvia, 2020–2022.

Nutrient	Range			Mean ± SE			CV, %
N	2.00	-	45.50	18.12	±	1.71	69.48
P	2.00	-	14.70	5.92	±	0.49	60.79
K	18.90	-	134.05	65.63	±	4.61	51.57
Ca	61.42	-	1027.04	426.27	±	34.02	58.65
Mg	40.13	-	281.54	107.39	±	6.02	41.21
S	3.50	-	33.78	6.39	±	0.62	71.58
Fe	13.05	-	373.55	111.55	±	8.09	53.27
Cu	0.25	-	1.60	0.64	±	0.04	41.52
Zn	1.23	-	22.58	4.89	±	0.47	70.60
Mn	0.35	-	31.36	3.87	±	0.68	129.89
B	0.10	-	0.60	0.22	±	0.02	54.52
Mo	0.02	-	0.07	0.03	±	0.00	29.27
pH_KCl_	2.68	-	4.25	2.94	±	0.04	14.48
EC	0.15	-	0.68	0.35	±	0.02	36.22

**Table 3:** Eigenvector values of principal component analysis of peat samples describing correlations with the first three components.

Variable	PC1	PC2	PC3
P	−0.286	−0.402	0.228
K	−0.461	−0.237	0.276
Ca	−0.234	0.496	0.033
Cu	−0.019	0.486	0.275
Zn	−0.448	0.131	0.115
Mn	−0.332	0.316	−0.356
pH_KCl_	0.175	0.391	0.538
EC	−0.470	−0.087	0.253
Organic matter	0.298	−0.153	0.549
Explained variance	34.4%	24.1%	12.2%

**Table 4:** (**A**) Nutrient concentration in air-dried cloudberry leaves in Latvia, 2020-2022; (**B**) sufficiency ranges for different berry leaves.

A								B			
Elements	Mean ± SE			CV, %	Range			Caneberries [28]	Strawberries [28]	Highbush Blueberries [29]	American Cranberries [30]
N, %	2.46	±	0.04	11.98	1.90	–	3.00	2.3–3.0	2.5–3.0	1.5–2.0	0.8–1.5
P, %	0.15	±	0.01	35.49	0.08	–	0.27	0.19–0.45	0.15–0.3	0.15–0.3	0.15–0.3
K, %	1.16	±	0.03	21.97	0.75	–	1.75	1.3–2.0	1.0–2.0	0.35–0.70	0.30–0.70
Ca, %	0.50	±	0.02	35.76	0.15	–	0.90	0.6–2.0	1.0–2.0	0.40–0.80	0.5–0.8
Mg, %	0.52	±	0.02	24.59	0.26	–	0.72	0.3–0.6	0.2–0.5	0.12–0.30	0.15–0.30
S, %	0.14	±	0.01	28.66	0.07	–	0.23	0.1–0.2	0.11–0.4	0.10–0.25	0.10–0.25
Fe, mg kg^−1^	63.75	±	2.72	31.31	34.67	–	129.00	60–250	60–200	60–150	60–150
Cu, mg kg^−1^	6.12	±	0.43	52.09	2.50	–	17.20	15–50	6–20	6–12	6–12
Zn, mg kg^−1^	71.37	±	2.34	24.13	43.61	–	121.00	6–20	20–50	15–60	20–80
Mn, mg kg^−1^	265.66	±	18.49	51.14	50.95	–	660.00	50–300	50–650	25–100	25–100
B, mg kg^−1^	17.57	±	0.77	31.99	8.00	–	29.00	30–70	25–45	20–60	20–60
Mo, mg kg^−1^	0.35	±	0.03	63.53	0.10	–	0.75	na	na	0.5–5	0.5–5

Sufficiency ranges for other berry leaves proposed by Strik et al. [28] and Nollendorfs [29,30]. na—no data.

**Table 5:** Pearson’s correlation coefficients between nutrient concentrations in soil and cloudberry leaves in Latvia.

Soil	**Plant**												
		**N**	**P**	**K**	**Ca**	**Mg**	**S**	**Fe**	**Cu**	**Zn**	**Mn**	**B**	**Mo**
	N	−0.025	0.003	0.070	0.053	0.288	−0.044	−0.135	−0.161	−0.269	0.166	−0.005	−0.235
	P	−0.076	0.127	0.033	0.132	0.084	0.001	−0.031	−0.072	0.321	0.457	−0.249	0.073
	K	−0.189	0.036	0.059	0.116	−0.151	−0.250	0.097	−0.181	0.161	0.349	−0.052	0.196
	Ca	−0.187	0.006	−0.227	−0.201	−0.343	−0.018	−0.085	0.179	−0.099	0.006	−0.252	0.254
	Mg	−0.093	0.070	−0.285	−0.105	−0.015	0.075	−0.156	0.020	−0.111	−0.086	−0.242	−0.039
	S	0.257	0.300	0.057	−0.132	−0.085	−0.057	−0.130	0.055	−0.055	0.013	−0.099	0.175
	Fe	−0.076	0.085	−0.100	−0.081	−0.195	−0.088	−0.095	−0.064	−0.118	0.351	−0.226	0.119
	Cu	−0.264	−0.088	−0.044	−0.373	−0.213	0.358	−0.209	0.177	−0.341	−0.042	−0.345	−0.196
	Zn	0.098	0.143	0.079	−0.028	−0.074	−0.266	0.004	−0.183	−0.110	0.047	0.062	−0.069
	Mn	−0.131	−0.110	−0.277	0.101	0.218	−0.192	0.018	−0.112	0.024	0.309	0.128	0.141
	B	−0.024	0.229	0.196	0.028	−0.099	−0.087	−0.099	0.177	−0.008	0.088	−0.076	0.294
	Mo	0.063	0.238	−0.084	0.003	−0.247	0.013	−0.077	0.260	0.277	0.206	−0.193	0.312

Statistically significant positive correlations are marked in beige, negative in blue (*n* = 54, *r* > 0.264, *p* = 0.05).

**Table 6:** Date of sample-gathering and corresponding period of the vegetation season during field survey of wild cloudberry stands in Latvia.

Sample Collection Period	2020	2021	2022
Before Flowering	Ruksi bog (8 June) Great Pelecare bog (9 June)	Lilaste (2 June)	
Flowering/Fruiting Season	Ramuli (6 July)	Medema bog (11 July)	Cena Moorland (14 June) Raganu bog (14 June) Melnezers (14 June) Stunisu Lake (6 July) Black Lake mire (6 July)
After Fruiting	Kaigu bog (15 August) Berzu bog (23 August) Skrebeli bog (24 August)	Salaspils bog (27 July) Great Kangari Lake (5 August)	Veikenieki bog (3 August) Dzelves bog (29 August) Lauga bog (29 August)

## Data Availability

All data reported here is available from the authors upon request.
